# Choledochal Villous Adenoma With Intrahepatic Bile Duct Stones: A Case Report

**DOI:** 10.1002/ccr3.73126

**Published:** 2026-07-10

**Authors:** Hao Li, Shaohua Sun, Wenbo Zhou

**Affiliations:** ^1^ Department of Hepatobiliary and Pancreatic Surgery, Sinopharm Dongfeng General Hospital Hubei University of Medicine Shiyan Hubei People's Republic of China

**Keywords:** case report, choledochal villous adenoma, extrahepatic biliary adenoma, hepatoportal tumor, high‐grade intraepithelial neoplasia, roux‐en‐Y choledochojejunostomy

## Abstract

This report describes a 58‐year‐old female with prior biliary surgery who presented with a choledochal villous adenoma and intrahepatic stones. The lesion showed high‐grade intraepithelial neoplasia, indicating malignant potential. She underwent left hemihepatectomy, choledochectomy, and Roux‐en‐Y hepaticojejunostomy, with no recurrence at 4‐month follow‐up.

## 1 Introduction

Villous adenomas of the extrahepatic bile duct are exceedingly rare, with a well‐documented tendency for malignant transformation into adenocarcinoma [1]. Unlike colorectal villous adenomas, their biliary counterpart is poorly characterized in the literature. This case report presents the diagnosis and surgical management of a patient with a choledochal villous adenoma complicated by intrahepatic bile duct stones, highlighting clinical decision‐making and histopathological features.

## 2 Case History/Examination

A 58‐year‐old female presented with epigastric discomfort for 1 week. Her past surgical history is summarized in Table 1.

**TABLE 1:** Timeline of prior biliary interventions.

Time prior to current admission	Procedure
> 4 years	Laparoscopic left lobectomy + choledochotomy with cholangioscopy + T‐tube drainage + cholecystectomy (for intrahepatic and extrahepatic stones)
2 years	ERCP with endoscopic papillary muscle incision lithotripsy + endoscopic nasobiliary drainage (for choledochal stones; papillary tumor suspected)

### 2.1 Physical Examination and Laboratory Findings

Physical examination: abdomen flat and soft, no tenderness or masses, liver and spleen not palpable, normal bowel sounds. Abnormal laboratory results on admission: total bilirubin 23.9 μmol/L (elevated), direct bilirubin 12.4 μmol/L, ALT 90 U/L (elevated), AST 47 U/L (elevated). Other liver function tests and routine blood counts were within normal limits.

### 2.2 Imaging Findings

**FIGURE 1:**
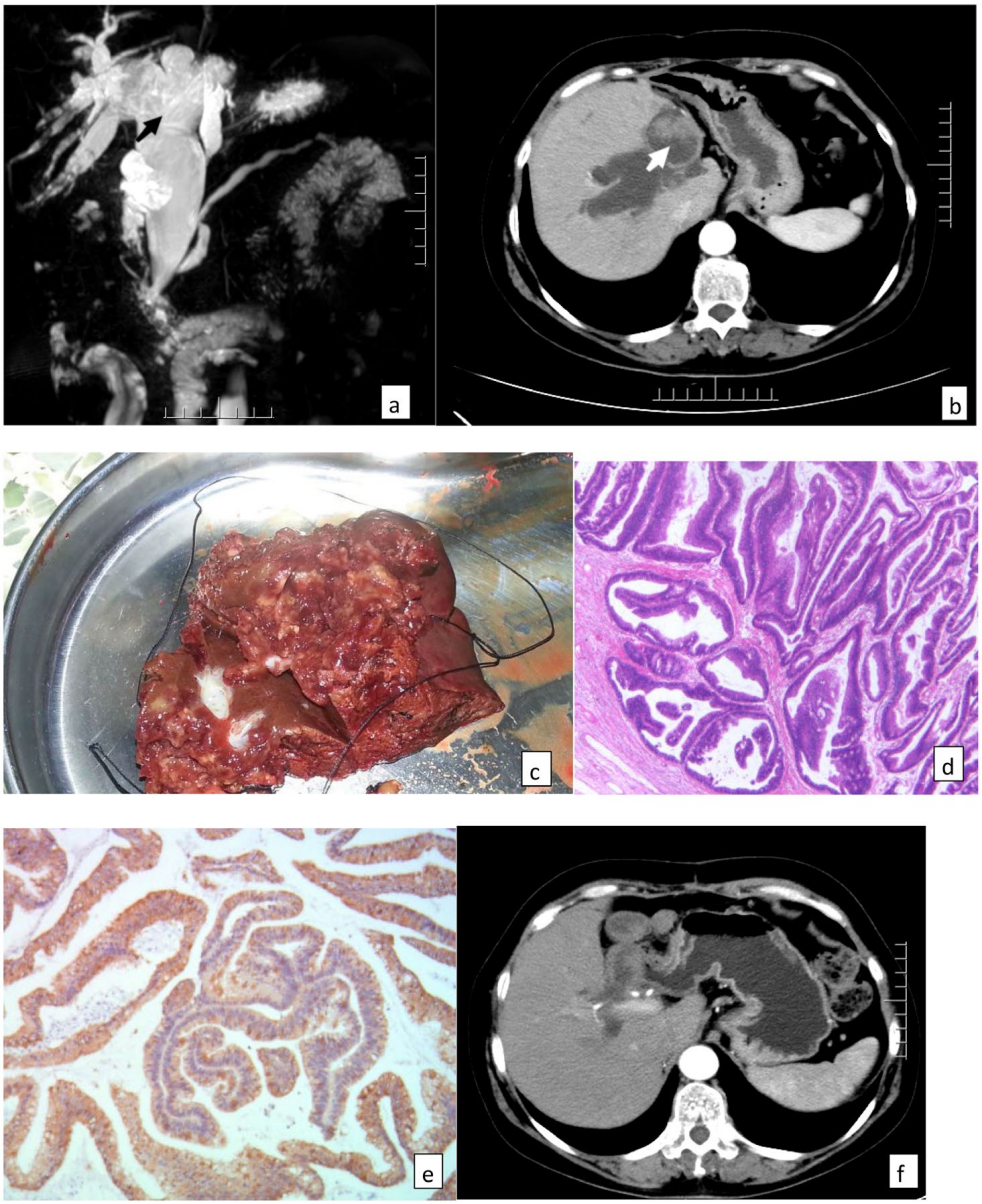
Preoperative imaging, gross pathology, histopathology, and postoperative follow‐up. (a) Preoperative MRCP: Dilated bile ducts and residual flocculent shadow in left hepatic duct. (b) Contrast‐enhanced CT: Flocculent soft tissue in dilated hilar bile ducts. (c) Gross left hemiliver specimen. (d) Histopathology (HE ×20): Tubulovillous adenoma (grade II–III) with high‐grade intraepithelial neoplasia; hepatocellular edema and focal lymphocytic infiltration. (e) Immunohistochemistry (×40): Diffuse CKpan positivity. (f) Four‐month postoperative contrast‐enhanced CT: Left lobe resection, bilioenteric and enteroenteric anastomoses, right intrahepatic duct dilation with mild pneumobilia; no recurrence.

MRCP (Figure 1a): marked intrahepatic and extrahepatic bile duct dilatation; right hepatic lobe stone with mild cholangitis; residual flocculent shadow in left hepatic duct. Contrast‐enhanced CT (Figure 1b): flocculent soft tissue within dilated hilar bile ducts, suspicious for neoplasm (possible intraductal papillary mucinous neoplasm). Also noted: right intrahepatic duct hyperdense shadow and small amount of hilar pneumobilia.

## 3 Differential Diagnosis, Investigations and Treatment

### 3.1 Differential Diagnosis

Preoperative diagnosis: bile duct lesion; choledocholithiasis with cholangitis; status post left lateral lobectomy, cholecystectomy, choledochotomy, and ERCP; hypertension. The differential diagnosis was narrowed to: intraductal papillary neoplasm of the bile duct (IPNB—low‐grade malignancy, requires histopathology); cholangiocarcinoma (suspected if infiltrative growth or lymph node metastasis); choledocholithiasis with cholangitis (may cause dilation and wall thickening but no intraluminal mass); and benign biliary stricture (from prior surgery/inflammation, smooth narrowing without filling defect). Definitive diagnosis relies on histopathology and immunohistochemistry.

### 3.2 Investigations and Treatment

Preoperative optimization included infection control (combined antibiotics), hepatoprotection (magnesium isoglycyrrhizinate), nutritional support (enteral nutrition for chronic disease‐related wasting), and comorbidity management (cardiology consultation to adjust antihypertensive regimen). A preoperative MDT (hepatobiliary surgery, anesthesiology, radiology, pathology) concluded that the neoplasm was likely located in the left hepatic duct involving the hilum, and surgery was indicated. The patient underwent left hemihepatectomy + choledochectomy + Roux‐en‐Y hepaticojejunostomy. Intraoperative findings: dilated left intrahepatic and common bile ducts; palpable tumor ~5 cm in diameter, soft, reduced mobility, unclear borders. Total blood loss: ~200 mL. Specimen gross pathology (Figure 1c): left hepatic duct 3.0 cm length, 3.5 cm diameter; intraluminal mass 3.0 × 3.0 × 2.5 cm, gray‐reddish, soft, ill‐defined. Dilated intrahepatic ducts (0.5–1.3 cm) with stones. Common bile duct segment: 4.0 × 1.5 cm; no lymph nodes in surrounding adipose tissue.

### 3.3 Histopathology and Immunohistochemistry

Diagnosis (Figure 1d,e): tubulovillous adenoma (grade II–III) with high‐grade intraepithelial neoplasia (HGIN). Surgical margin: left hepatic duct margin positive for HGIN. Associated findings: pigmented gallstones attached to duct wall; dilated left hemiliver ducts with stones. Immunohistochemistry results are summarized in Table 2.

**TABLE 2:** Summary of immunohistochemical markers.

Marker	Result	Interpretation
CKpan	+	Confirms epithelial origin
p53	Variable intensity	Suggests aberrant expression in HGIN
Ki‐67	60% positive	High proliferative index
SMA, Desmin	−	No smooth muscle differentiation
CD34, D2‐40	−	No vascular or lymphatic tumor thrombus
HBsAg, HBcAg	−	No hepatitis B infection

## 4 Conclusion and Results (Outcome and Follow‐Up)

The patient recovered uneventfully and was discharged on postoperative Day 14. At 4‐month follow‐up (contrast‐enhanced CT, Figure 1f): postoperative changes (left lobe resection, bilioenteric and enteroenteric anastomoses); right hepatic lobe bile duct widening with small amount of pneumobilia; no evidence of tumor recurrence. The patient was asymptomatic and in good general condition.

## 5 Discussion

Villous adenoma of the bile duct is an extremely rare neoplasm with a documented risk of progression to adenocarcinoma [1]. Its etiology remains unclear, but chronic irritation from gallstones and inflammation likely contributes to epithelial hyperplasia and papillary change. Genetic alterations (e.g., BRAF V600E) have been implicated in some biliary adenomatosis cases [2]. Recent single‐cell studies highlight that tumor heterogeneity and perineural invasion (PNI) in cholangiocarcinoma involve GFAP+ dedifferentiated Schwann cells [3]; similar mechanisms may operate in villous adenoma during malignant transformation. Intraductal papillary neoplasm of the bile duct (IPNB), a recognized precursor lesion, shows cancer cells originating within the duct lumen before invading liver parenchyma [4]. Animal models demonstrate that FGF10‐induced ERK signaling can recapitulate IPNB progression [5]. In our patient, repeated episodes of choledocholithiasis and cholangitis likely provided the chronic irritation that led to villous adenoma formation. While often asymptomatic, hilar lesions can cause obstructive jaundice. Common symptoms include intermittent pain, dyspepsia, weight loss, nausea, vomiting, and fever. Imaging studies show bile duct dilation in about 60% of cases [3]. Brush cytology is a common initial diagnostic technique [4], but definitive diagnosis requires histopathological evaluation. Complete surgical resection is the treatment of choice for resectable lesions. ERCP may palliate symptoms but carries a risk of recurrence [5]. Surgery is indicated if malignancy is suspected or tumor > 2 cm. In our patient, the tumor involved the middle‐upper common bile duct with residual left intrahepatic stones, prompting left hemihepatectomy + choledochectomy + Roux‐en‐Y hepaticojejunostomy, which achieved an excellent outcome. For distal common bile duct lesions where malignancy cannot be excluded, pancreaticoduodenectomy is appropriate. Some authors also recommend local resection with hepaticoduodenal ligament lymphadenectomy for middle‐lower duct lesions with malignant potential [6]; liver transplantation may be considered for advanced disease. Short‐term follow‐up studies indicate low recurrence rates after complete resection [7]. Our patient showed no recurrence at 4 months, but due to the presence of high‐grade intraepithelial neoplasia and a positive ductal margin (left hepatic duct), long‐term surveillance is critical. We recommend annual contrast‐enhanced CT or MRCP with tumor markers (CA19‐9, CEA) for at least 5 years, and continued follow‐up thereafter given the risk of late recurrence or development of adenocarcinoma.

## 6 Teaching Points

1. Choledochal villous adenoma should be considered in patients with recurrent biliary symptoms, especially those with a history of choledocholithiasis or prior biliary surgery.
2. The combination of intrahepatic stones and a bile duct mass warrants thorough imaging and MDT discussion to rule out malignancy.
3. Complete surgical resection (including hepaticojejunostomy when needed) offers the best chance for cure even for histologically benign but premalignant tumors.
4. High‐grade intraepithelial neoplasia and positive margins necessitate long‐term surveillance (annual imaging for ≥ 5 years) due to risk of recurrence and progression.
5. Immunohistochemistry (Ki‐67, p53) and grading help stratify malignant potential and guide follow‐up intensity.

## Author Contributions

**Hao Li:** investigation, visualization, writing – original draft. **Shaohua Sun:** data curation, investigation, writing – review and editing. **Wenbo Zhou:** conceptualization, funding acquisition, project administration, supervision, writing – review and editing.

## Funding

The Guided Project of Education Department of Hubei Province (Grant No. B2024109); the Guided Project of Shiyan Municipal Bureau of Science and Technology (Grant No. 24Y155). The funders had no role in study design, data collection and analysis, decision to publish, or preparation of the manuscript.

## Consent

Written informed consent was obtained from the patient to publish this report in accordance with the journal's patient consent policy.

## Conflicts of Interest

The authors declare no conflicts of interest.

## Data Availability

The data that support the findings of this study are available from the corresponding author upon reasonable request.

## References

[1] A. Yadav and S. Nundy , “Case Series of Non‐Ampullary Duodenal Adenomas,” Annals of Medicine and Surgery 69 (2021): 102730, 10.1016/j.amsu.2021.102730. 34484721 PMC8408424

[2] J. Augustin , J. Calderaro , and A. Pujals , “BRAF‐Associated Bile Duct Adenomatosis: A New Entity?,” Histopathology 77 (2020): 160–161. 32367668 10.1111/his.14125

[3] Z. Zu , C. Zhang , J. Shi , et al., “Single‐Cell Analysis Reveals That GFAP+ Dedifferentiated Schwann Cells Promote Tumor Progress in PNI‐Positive Distal Cholangiocarcinoma via Lactate/HMGB1 Axis,” Cell Death & Disease 16, no. 1 (2025): 215, 10.1038/s41419-025-07543-x. 40148311 PMC11950304

[4] K. Chiablaem , A. Jinawath , J. Nuanpirom , et al., “Identification of RNF213 as a Potential Suppressor of Local Invasion in Intrahepatic Cholangiocarcinoma,” Laboratory Investigation 104, no. 7 (2024): 102074, 10.1016/j.labinv.2024.102074. 38723854

[5] H. Tomita , K. Tanaka , A. Hirata , et al., “Inhibition of FGF10‐ERK Signal Activation Suppresses Intraductal Papillary Neoplasm of the Bile Duct and Its Associated Carcinomas,” Cell Reports 34, no. 8 (2021): 108772, 10.1016/j.celrep.2021.108772. 33626352

[6] S. Seyfried , G. Kähler , S. Belle , et al., “Endoscopic Papillectomy or Pancreaticoduodenectomy for Ampullary Lesions: A Single Center Retrospective Cohort Study,” Scandinavian Journal of Gastroenterology 57, no. 11 (2022): 1381–1389. 35723057 10.1080/00365521.2022.2088243

[7] S. Muro , H. Kato , A. Matsumi , et al., “The Long‐Term Outcomes of Endoscopic Papillectomy and Management of Cases of Incomplete Resection: A Single‐Center Study,” Journal of Gastrointestinal Surgery 25, no. 5 (2021): 1247–1252. 32583320 10.1007/s11605-020-04532-7

